# The Balance between Recombination Enzymes and Accessory Replicative Helicases in Facilitating Genome Duplication

**DOI:** 10.3390/genes7080042

**Published:** 2016-07-29

**Authors:** Aisha H. Syeda, John Atkinson, Robert G. Lloyd, Peter McGlynn

**Affiliations:** 1 Department of Biology, University of York, Wentworth Way, York YO10 5DD, UK; aisha.syeda@york.ac.uk; 2 School of Medical Sciences, Institute of Medical Sciences, University of Aberdeen, Foresterhill, Aberdeen AB25 2ZD, UK; j.d.atkinson@gmx.com; 3 Centre for Genetics and Genomics, University of Nottingham, Queen’s Medical Centre, Nottingham NG7 2UH, UK; bob.lloyd@nottingham.ac.uk

**Keywords:** genome stability, repair, replication, RNA polymerase

## Abstract

Accessory replicative helicases aid the primary replicative helicase in duplicating protein-bound DNA, especially transcribed DNA. Recombination enzymes also aid genome duplication by facilitating the repair of DNA lesions via strand exchange and also processing of blocked fork DNA to generate structures onto which the replisome can be reloaded. There is significant interplay between accessory helicases and recombination enzymes in both bacteria and lower eukaryotes but how these replication repair systems interact to ensure efficient genome duplication remains unclear. Here, we demonstrate that the DNA content defects of *Escherichia coli* cells lacking the strand exchange protein RecA are driven primarily by conflicts between replication and transcription, as is the case in cells lacking the accessory helicase Rep. However, in contrast to Rep, neither RecA nor RecBCD, the helicase/exonuclease that loads RecA onto dsDNA ends, is important for maintaining rapid chromosome duplication. Furthermore, RecA and RecBCD together can sustain viability in the absence of accessory replicative helicases but only when transcriptional barriers to replication are suppressed by an RNA polymerase mutation. Our data indicate that the minimisation of replisome pausing by accessory helicases has a more significant impact on successful completion of chromosome duplication than recombination-directed fork repair.

## 1. Introduction

The replication machineries of all organisms encounter many potential barriers whilst duplicating their genomes, presenting a major challenge to the maintenance of genetic stability [1,2]. These barriers include damage to the template, non-B form DNA structures, topological strain and proteins bound to the DNA. Transcription provides both a topological challenge to DNA replication due to the over- and underwinding ahead of and behind an advancing RNA polymerase [3,4] and substantial nucleoprotein barriers to fork movement due to their very high affinity [5]. Individual nucleoprotein complexes may have a low probability of halting a replication fork but the large number of barriers encountered creates a substantial risk of failure to complete high fidelity genome duplication [6,7]. Replisomes paused at these barriers retain activity but this activity is lost as a function of time [8,9,10,11]. There is thus a window of opportunity for removal or bypass of the barrier and resumption of replication by the paused replisome. If clearance or bypass of the barrier does not occur prior to loss of paused replisome function then the replication machinery must be reloaded back onto the chromosome to facilitate completion of genome duplication [1]. Given the importance of completing high fidelity genome duplication, all organisms have evolved mechanisms to underpin replisome movement by facilitating the restart of paused replisomes and by reconstituting an active replication fork after loss of paused replisome activity.

Upon encountering a barrier, the replisome itself can clear or bypass certain types of obstacle. Forks paused at single-stranded DNA lesions may bypass the lesion by repriming replication downstream of the barrier, allowing resumption of replication at the cost of a gap in one of the nascent DNA strands [12,13,14,15,16]. However, bypass does not approach 100% efficiency, implying that replisomes encountering many lesions have a significant probability of losing activity [15]. Specialised translesion DNA polymerases can also replicate across such lesions under certain circumstances but often at the cost of errors in base incorporation [17,18,19]. The replisome is also capable of displacing proteins bound to the template DNA [20,21,22], a property that reflects the ability of helicases to disrupt the non-covalent bonding between proteins and DNA [23,24]. Forks do also pause stochastically at protein-DNA complexes but the paused replisome may resume movement if the blocking protein dissociates from the DNA prior to loss of activity of the paused replisome [20]. However, the barriers posed by the many protein-DNA complexes found within a chromosome, especially those associated with transcription, appear to be too numerous and/or too long-lived for the replisome itself to deal with during the course of genome duplication. The *Saccharomyces cerevisiae* RRM3 helicase minimises fork blockage at non-histone protein-DNA complexes and is required for normal rates of fork movement [25,26,27,28]. Similarly, the *E. coli* Rep helicase promotes fork movement through nucleoprotein complexes and its absence results in at least a twofold increase in the time needed to replicate a chromosome [22,29,30,31]. This increase in the time needed for genome duplication reflects the function of Rep in minimising the frequency and/or duration of replisome pausing at protein-DNA complexes, the primary sources of replication pausing in *E. coli* [6]. The *Bacillus subtilis* helicase PcrA, a homologue of *E. coli* Rep, also facilitates replication of transcribed DNA in vivo [32], indicating conservation of this function across evolutionarily very divergent organisms. Both RRM3 and Rep also associate physically with components of their respective replisomes [22,28,33]. In *E. coli* the physical association between Rep and the primary replicative helicase DnaB promotes cooperative DNA unwinding and nucleoprotein complex removal by the two helicases [22,34,35]. However, although *B. subtilis* PcrA is essential for viability [36], neither *S. cerevisiae* RRM3 nor *E. coli* Rep are needed for viability [37,38]. These enzymes are now considered to be accessory replicative helicases that minimise replisome pausing along protein-bound DNA, whilst the primary replicative helicase is responsible for template DNA unwinding and acts as a hub for replisome organisation [39,40].

The above mechanisms reduce the probability of loss of function of replisomes encountering barriers that can be either cleared or bypassed. These mechanisms therefore rely on retention of function of paused replisomes. However, the large number of barriers encountered by replisomes means that there is still a significant risk of a replisome pausing at a barrier and losing function prior to bypass or clearance of the barrier [1,7]. This is a particular problem with arrays of transcription complexes on highly transcribed genes [30,41,42,43,44,45]. Blockage of a fork and loss of replisome function demands reloading of the replication machinery to complete genome duplication, even when multiple origins exist on the same chromosome [46]. Generation of a DNA structure onto which the replication machinery can be reloaded may require substantial remodelling of the fork DNA by a combination of exonucleases, endonucleases and helicases to facilitate replisome reloading [2,7]. Such processing may also require strand exchange proteins either to reintegrate double-stranded DNA ends generated by fork processing, to repair single-stranded DNA gaps or to catalyse replication fork regression [1,47,48]. Strand exchange proteins might also promote blocked fork stabilisation, inhibiting extensive degradation of nascent DNA via occlusion of nucleases [49,50,51]. The bacterial strand exchange protein RecA minimises degradation of nascent DNA in *E. coli* cells exposed to UV light [52]. This minimisation also requires RecFOR, factors that promote RecA loading onto ssDNA gaps rather than dsDNA ends, together with RecJ exonuclease and RecQ helicase [52,53].

The general view now is that a major role of recombination enzymes, if not their primary purpose, is to underpin replication fork movement [54]. The importance of such enzymes is illustrated by the extensive DNA degradation in *recA* mutant cells [55]. This degradation is catalysed by RecBCD, a helicase and exonuclease that unwinds and degrades dsDNA ends [55,56]. Degradation of both DNA strands by RecBCD is rapid and processive but recognition of a specific DNA sequence, a χ site, within the DNA inhibits degradation of the 3’ ended strand and promotes loading of RecA onto this strand [56]. However, degradation continues in the absence of RecA, with RecBCD being able to degrade an entire chromosome arm [55,57]. Some blocked forks may also undergo regression and extrude a dsDNA arm which may be degraded by RecBCD in the absence of RecA, effectively destroying the extruded arm of the fork and regenerating a fork structure onto which the replisome can be reloaded [58].

Targeting of blocked forks by recombination enzymes comes at the cost of genome rearrangements [59,60]. This genetic instability is a particular problem at highly transcribed genes due to the density of transcribing RNA polymerases and the consequent high probability of fork pausing, loss of replisome function and the need to process the DNA via recombination enzymes to reload the replisome [2,61,62,63]. Moreover, loss of factors that minimise stalled and backtracked transcription complexes increase the dependence of *E. coli* cells on recombination enzymes [42]. The absence of accessory replicative helicases that restart paused forks also exacerbates the pathological effects of replication-associated recombination [64,65]. Thus, *E. coli* Rep limits harmful RecA loading at blocked forks [64]. Increasing the probability of fork pausing or of paused forks losing function therefore results in an increased need for recombination enzymes to underpin genome duplication.

Such is the potentially catastrophic effect of unregulated strand exchange that organisms have also evolved other means of limiting binding of strand exchange proteins to ssDNA. Turnover of strand exchange protein-ssDNA filaments by helicases is a key mechanism employed in both bacteria and eukaryotes to limit homologous recombination [66] with UvrD helicase performing this task in *E. coli* [67].

Accessory helicases target paused, active replisomes whereas recombination enzymes process blocked forks that no longer retain an active replisome. The substrates for these classes of enzymes are therefore very different. *S. cerevisiae rrm3* mutant cells are viable but require replication, repair and checkpoint genes for normal growth [68,69,70]. Similarly, *E. coli* cells lacking either RecBCD or Rep are viable but cells lacking both are inviable [71,72]. In contrast, the viability of *E. coli* *recA rep* mutant cells indicates that processing of inactivated forks does not necessarily require strand exchange [71]. This viability reflects the ability of RecBCD to degrade partly replicated chromosomes when RecA is absent [55,58]. Indeed, RecBCD but not RecA, is essential for viability in the presence of an inverted and highly expressed ribosomal operon [73]. It should be borne in mind, though, that RecBCD activity in the presence of RecA results in loading of RecA onto the single-stranded DNA generated by RecBCD, strand exchange and priming of DNA replication via a D-loop recombination intermediate [7,56,74].

*E. coli* Δ*rep* mutant cells are viable in part because a homologous helicase, UvrD, can also promote fork movement along protein-bound DNA and, thus, compensate partially for the absence of Rep [22,30]. Single deletion mutants are therefore viable whereas Δ*rep* Δ*uvrD* mutants are not [75]. The lack of full compensation may be because UvrD, unlike Rep, does not interact with the replisome via DnaB [22]. DinG helicase has also been implicated in resolving conflicts between replication and transcription in concert with Rep and/or UvrD [30]. However, the mechanistic basis of this interplay remains unclear with no direct evidence that DinG displaces proteins ahead of advancing replication forks [40]. It is clear, though, that Δ*rep* mutant cells but neither Δ*uvrD* nor Δ*dinG* mutants exhibit a significant extension of the time needed to replicate the chromosome [29,31]. This Rep-specific defect indicates that Rep rather than UvrD or DinG plays a key role in maintaining rapid fork movement.

Δ*rep* Δ*uvrD* double mutant inviability can be suppressed by growth of Δ*rep* Δ*uvrD* mutant cells on minimal medium, conditions under which levels of transcription are reduced as compared with rich medium growth [22,30]. Δ*rep* Δ*uvrD* inviability on rich medium is also partially suppressed by two classes of mutation. One class of mutants harbour mutations in *spoT* which leads to elevated concentrations of the signalling molecule (p)ppGpp [22]. (p)ppGpp binds to RNA polymerase and inhibits initiation of transcription of many genes including the *rrn* operons in *E. coli*, the source of half of all transcription under rapid growth conditions, and also destabilises stalled transcription complexes [42,76]. These effects may reduce the number of replicative barriers presented by transcription. Elevated (p)ppGpp also reduces replication elongation rates which might result in fewer collisions between transcription and replication, although the elongation rate is only modestly affected in *E. coli* [77]. The second class of mutations reside in the structural genes for RNA polymerase [22,30,78]. These mutations may suppress via different mechanisms depending on the nature of the mutation but may act in a similar manner to elevated (p)ppGpp [42,78,79] and/or reduce the extent of backtracking of paused RNA polymerases [80]. For example, the Δ*rep* Δ*uvrD* double mutant suppressor rpoB*35 allows cells unable to synthesise (p)ppGpp to grow on minimal medium, a so-called stringent phenotype which indicates that rpoB*35 phenocopies elevated (p)ppGpp [22,79]. rpoB*35 may also destabilise transcription complexes stalled by nucleotide starvation or DNA lesions [42] although this has been questioned and data presented indicating this mutant RNA polymerase has a reduced probability of backtracking [80].

Another class of mutations provide weaker suppression of Δ*rep* Δ*uvrD* double mutant inviability. These suppressors have defects in the RecA loading factors RecF, RecO or RecR or in RecJ exonuclease or RecQ helicase, all of which facilitate RecA loading onto single-stranded DNA gaps [22,30,81,82]. This suppression may reflect the potential for toxic levels of RecFORQJ-dependent strand exchange by RecA at blocked forks [81,83,84]. In Δ*rep* Δ*uvrD* double mutant cells, elevated fork pausing together with the lack of UvrD-catalysed disruption of RecA-ssDNA filaments may explain why ablation of RecFORQJ-dependent RecA loading partially suppresses Δ*rep* Δ*uvrD* double mutant inviability. However, UvrD cannot counter the adverse effects of RecAFORQJ in Δ*rep* mutant cells [64] implying that lack of RecA-ssDNA turnover is not the primary reason why RecAFORQJ is so toxic in Δ*rep* Δ*uvrD* mutant cells.

The relative importance of accessory helicases and recombination enzymes for genome duplication remains unclear. *E. coli* cells lacking RecA or RecBCD have reduced viability [85]. Cells bearing inverted *rrn* operons do not require Rep for viability but do require either RecBCD helicase/exonuclease or RecBCD helicase lacking exonuclease activity plus RecA [30,73]. These data suggest that RecBCD and RecA have a more important role in replicating the chromosome than Rep. However, during normal growth without inverted highly expressed operons there is insufficient recombination to require Holliday junction resolution for viability [86]. Only in the absence of Rep does this resolution become important for viability [64], consistent with Rep having a primary role in sustaining completion of chromosome replication.

Here we show that the known chromosome content defects of *recA* cells is driven primarily by transcription, mirroring the importance of transcriptional barriers to replication in the chromosome content defects of Δ*rep* mutant cells [6]. Both RecA and Rep therefore have roles in mitigating the impact of transcription on genome duplication. However, in contrast to Rep [31], neither RecA nor RecBCD play important roles in sustaining wild type chromosome duplication times. These data indicate that accessory helicases play a more significant role than recombination enzymes in sustaining rapid chromosome duplication. This view is supported by RecA and RecBCD being able to sustain viability in the absence of Rep and UvrD but only in the presence of an RNA polymerase mutation that alleviates transcriptional barriers to replication. Furthermore, both RecA and RecBCD are needed for this viability, indicating that RecBCD-catalysed DNA degradation in the absence of RecA loading does not provide an efficient means of sustaining chromosome duplication. We conclude that accessory helicases are more important than recombination enzymes for replicating the *E. coli* chromosome but that replicative barriers normally dealt with by accessory helicases can be surmounted by less efficient mechanisms via recombination enzymes.

## 2. Materials and Methods

### 2.1. Plasmids and Strains

Strains are listed in Supplementary Table S1 and were constructed using P1 transduction. pAM375, pAM383, pAM403 [64], pAM406 and pAM407 [22] are derivatives of pRC7 [87] and encode *recB^+^*, *recA^+^*, *rep^+^*, *recA^+^*, *recB^+^* and *uvrD^+^*, respectively. pAM406 was made by cloning an ApaI fragment carrying *recA^+^* from pAM383 [64] into the ApaI site of pAM375 [64]. N6618 is a derivative of MG1655 carrying a deletion of *recA* in which all but 42 bp at both the 5’ and 3’ end of the gene sequence has been replaced with a sequence encoding resistance to kanamycin. It was made using the protocols described [88].

### 2.2. Flow Cytometry

**Figure 1:**
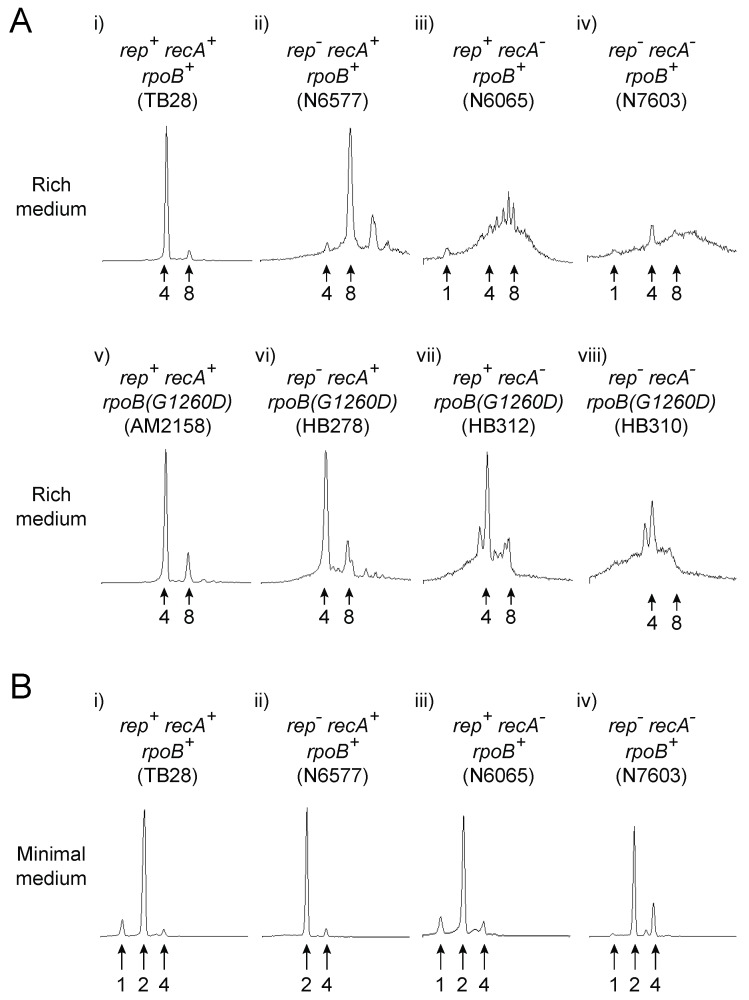
The chromosome content defects in the absence of Rep and RecA on rich medium are suppressed by an RNA polymerase mutation or by growth on minimal medium. (**A**) DNA content of the indicated strains grown to mid-logarithmic phase in LB medium as monitored by flow cytometry under run out conditions. The number of chromosome equivalents is indicated below; (**B**) DNA content of the strains used in (Figure 1A (i–iv)) grown to mid-logarithmic phase in minimal medium as monitored by flow cytometry under run out conditions.

**Figure 2:**
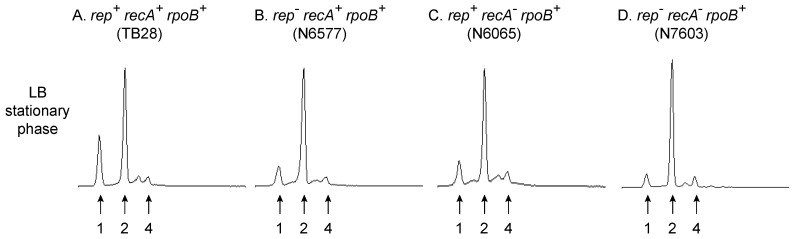
The chromosome content defects of *rep* and *recA* mutant cells at mid-logarithmic phase in rich medium are resolved by the time stationary phase is reached. Strains (**A**–**D**) are the same as those used in Figure 1A (i–iv).

**Figure 3:**
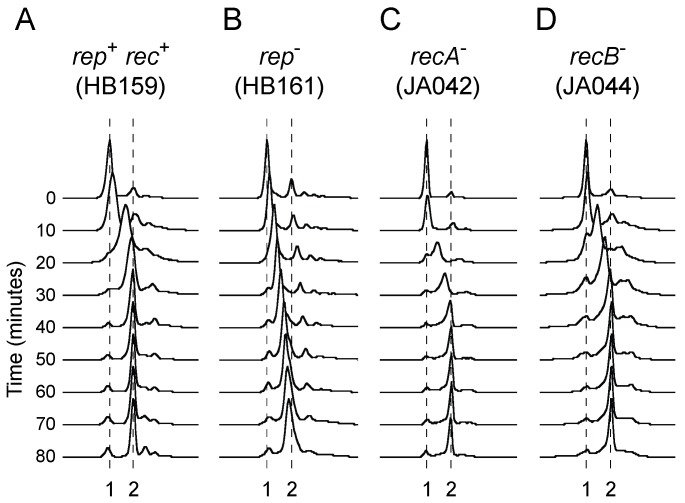
Chromosome duplication time is extended in *rep* but not *recA* or *recB* cells. (**A**–**D**) Flow cytometry profiles of the indicated strains in which initiation of chromosome duplication was synchronised at 42 °C by exploiting the presence of the temperature-sensitive *dnaA46* allele. Samples were analysed immediately after shifting the temperature from 42 °C to 30 °C (time 0). Cultures were then returned to 42 °C after 10 min. Samples were removed every 10 min after the temperature downshift. The number of chromosome equivalents is indicated below.

Flow cytometry to analyse DNA content in Figure 1 was performed on cells grown to mid-log phase in either LB or 56/2 salts minimal medium after treatment with rifampicin and cephalexin as described [6]. The DNA content of stationary phase cells (Figure 2) was performed in an identical manner except that cells were grown overnight prior to treatment with rifampicin and cephalexin. Flow cytometric analysis of chromosome duplication time (Figure 3) was performed as described [31].

### 2.3. Synthetic Lethality Assays

The ability of strains to form colonies upon loss of pRC7 derivatives was assessed as described [64]. After growth in the absence of ampicillin selection for pRC7 plasmids, cell were plated onto LB agar containing 120 μg/mL Xgal and 1 mM IPTG and incubated at 37 °C for 48 h.

## 3. Results

### 3.1. Transcription Is a Major Cause of Chromosome Degradation in recA Cells

One key feature of *E. coli* cells lacking the strand exchange protein RecA in otherwise unperturbed cells is elevated levels of RecBCD-dependent chromosome degradation [55]. This degradation is manifest as formation of cells with a range of different numbers of chromosome equivalents as detected by flow cytometry [55] (see also Figure 1A (i,iii)).

Thus cells require strand exchange for normal chromosomal duplication even in the absence of elevated DNA damage or engineered nucleoprotein barriers. The trigger(s) for this enhanced degradation are unclear and so we tested whether this degradation is attributable to transcription. We employed flow cytometry under run-out conditions to monitor DNA content in cells harbouring either wild type RNA polymerase or a mutant form of the complex resulting from the *rpoB(G1260D)* allele [6,89] (Figure 1). *rpoB(G1260D)* displays the same phenotypes as rpoB*35 including a stringent phenotype, suppression of Δ*rep* Δ*uvrD* double mutant lethality and suppression of chromosome replication defects in Δ*rep* muant cells [6,89] Most wild type cells contain 4 chromosome equivalents after run out during logarithmic growth in rich medium in both *rpoB^+^* and *rpoB(G1260D)* cells [6,55] (also compare Figure 1A (i) with Figure 1A (v)). In contrast, Δ*rep* *rpoB^+^* cells lacking the accessory replicative helicase Rep contain 8 chromosomes due to the increased time needed to replicate the chromosome and hence more replication origin firings per cell cycle [6,29,90]. *rpoB(G1260D)* suppresses this Δ*rep* mutant phenotype by reducing replisome pausing, with most Δ*rep rpoB(G1260D)* cells having 4 rather than 8 chromosomes [6] (see also Figure 1A (v–vi)). We found that *rpoB(G1260D)* also substantially suppressed the broad spread of chromosome equivalents seen in *recA* mutant cells (Figure 1A, compare (iii) with (vii)). We also tested cells lacking both Rep and RecA. Δ*rep recA rpoB^+^* cells had a more severe defect in chromosome content as compared with the single mutants (Figure 1A, compare (iv) with (ii) and (iii)). There is therefore significant synergy between Rep and RecA function in maintaining chromosome duplication. However, *rpoB(G1260D)* still provided partial suppression of this severe defect (Figure 1A, compare (iv) and (viii)).

Suppression of chromosome replication defects by *rpoB(G1260D)* in cells lacking Rep, RecA or both enzymes is consistent with transcription being the primary driver of these defects. Replication-transcription conflicts can also be alleviated by growth of *rpoB^+^* strains in minimal medium [22,30]. We tested therefore whether the major differences in DNA content in wild type versus Δ*rep, recA* or Δ*rep recA* mutant cells seen in mid-logarithmic cells grown in rich medium were recapitulated in minimal medium. We found that the majority of *rpoB^+^* cells either with or without Δ*rep* and/or *recA* mutant alleles contained 2 chromosome equivalents when grown to mid-logarithmic phase in minimal medium (Figure 1B (i–iv)). Restricting growth rate reduces therefore the chromosomal defects caused by the absence of Rep and/or RecA (compare Figure 1A (i–iv) with Figure 1B (i–iv)) supporting our conclusion that transcription is a major cause of the perturbed chromosome content observed in the absence of Rep and/or RecA.

We also investigated the ability of Δ*rep recA* mutant cells to remain viable even when so few of the cells contain an integral number of chromosomes under run out conditions during logarithmic growth in LB (Figure 1A (iv)). Flow cytometric analyses of *rpoB^+^* strains grown to stationary phase in LB revealed that the absence of functional Rep and/or RecA had little impact on chromosome content with the majority of cells in all cases containing two chromosomes (Figure 2A–D). Thus even cells lacking both Rep and RecA can eventually complete chromosome duplication to allow formation of viable progeny. Any barriers to completion of chromosome duplication in the absence of Rep and RecA must eventually be cleared therefore and must not generate replication intermediates that cannot be resolved (compare Figure 2A,D). There is much evidence that RecBCD helicase/exonuclease provides such a mechanism to degrade blocked replication intermediates when RecA is not available to initiate strand exchange from RecBCD-generated ssDNA [58,71,72]. However, the inviability of *rep recB* double mutant cells [71] precludes direct analysis of absence of both Rep and RecBCD on chromosome content by flow cytometry.

### 3.2. Rapid Chromosome Duplication Has a Greater Requirement for Rep than for RecA

The above data do not address the relative importance of Rep and recombination enzymes in underpinning efficient fork movement. The time taken to replicate chromosomes during a single cell cycle was therefore estimated using flow cytometry in strains lacking either Rep or RecA. Upon synchronising replication initiation using the temperature-sensitive *dnaA46* allele, wild type cells take 40–50 min for their DNA content to increase from 1 to 2 chromosome equivalents but Δ*rep* cells take more than 80 min [31] (see also Figure 3A,B). This extended duplication time reflects the impact of nucleoprotein complexes on fork progression in the absence of Rep [22,30]. In contrast to Δ*rep* mutant cells, we found that the majority of *recA* mutant cells had completed genome duplication after 40–50 min (Figure 3C). We also tested the time taken for chromosome duplication in *recB* mutant cells. The requirement for either Rep or RecBCD for survival implies that one or the other of these enzymes provides an essential means of underpinning fork progression [71,72]. However, *recB* mutant cells had chromosome duplication times similar to those found in wild type and *recA* mutant cells (Figure 3D).

These data demonstrate that absence of either RecA or RecBCD does not lead to significant slowing of the mean time taken for replication forks to travel from *oriC* to the terminus region. Processing of blocked replication forks by either RecA or RecBCD is therefore not critical for rapid chromosome duplication.

### 3.3. Both RecA and RecBCD Are Needed in the Absence of Accessory Helicase Activity

**Figure 4:**
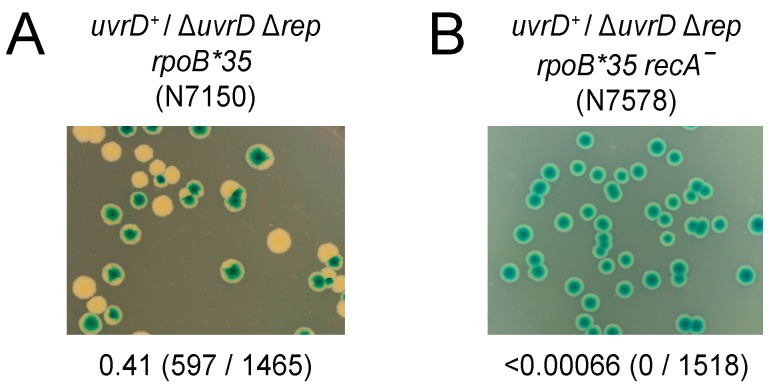
RecA is essential in the absence of Rep and UvrD on rich medium. (**A**,**B**) The ability to form colonies in the absence of RecA was monitored in the indicated strains on LB plates containing Xgal and IPTG. The parental strains contain pAM407 (pRC7*uvrD*) bearing both the *uvrD* gene and the *lac* operon and plasmidless cells give rise to white or segregated colonies due to loss of the *lac* operon. Fractions of white colonies are indicated below each panel and the actual number of white colonies and of total colonies are shown in parentheses.

The above data suggest Rep rather than RecA plays the dominant role in ensuring rapid genome duplication. It is clear, though, that transcription is a shared source of replicative defects in cells deficient in either Rep or RecA (Figure 1). However, the viability of *rep recA* double mutant cells [71] argues against a requirement for either Rep or RecA to overcome transcriptional barriers to replication. Interpretation of *rep recA* double mutant viability is complicated, though, since UvrD compensates partially for the absence of Rep accessory helicase function [22,30]. The requirement for RecA was tested therefore in the absence of both Rep and UvrD by using a Δ*rep* Δ*uvrD* double mutant strain rendered viable by the rpoB*35 allele via a reduction in replication-transcription conflicts [22,42,79]. A plasmid loss assay was employed in which retention of a highly unstable complementing plasmid can be monitored by blue/white screening [87]. Δ*rep* ΔuvrD rpoB*35 cells can lose pRC7*uvrD* on LB as indicated by the formation of white plasmidless colonies [22] (see also Figure 4A). In contrast, Δ*rep* ΔuvrD rpoB*35 recA cells could not lose pRC7*uvrD* on LB indicating that RecA is essential for viability in a Δ*rep* ΔuvrD rpoB*35 strain under rapid growth conditions (Figure 4, compare B with A). Thus even when transcriptional barriers to replication are reduced by the rpoB*35 allele there remains a requirement for either accessory helicase function or RecA.

**Figure 5:**
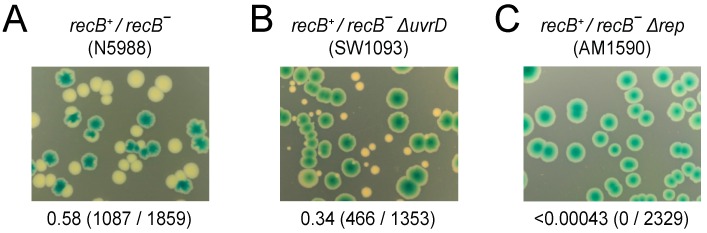
*uvrD recB* double mutant cells are viable but have a growth defect. (**A**–**C**) The ability of the indicated strains to lose pAM375 (pRC7*recB*) was monitored on LB Xgal IPTG plates.

The corollary of Δ*rep* ΔuvrD rpoB*35 recA inviability is that RecBCD is unable to maintain viability without RecA in this context. This requirement for RecA is in contrast to the viability of *rep recA* double mutant cells versus the inviability of *rep recB* double mutants in a *rpoB^+^* background [71]. This differential requirement in *uvrD^+^ rep^−^* cells reflects the generation of double-stranded DNA ends by regression of blocked replication forks and the need for RecBCD to process these ends [58]. Processing can occur either by loading of RecA followed by strand exchange or, in the absence of RecA, RecBCD-catalysed degradation of the dsDNA end to regenerate a fork structure [58]. The viability of *uvrD recB* double mutant strains is less certain. Absence of UvrD-catalysed removal of RecFOR-loaded RecA from blocked forks may lead to an increased need for RecBCD-dependent repair of dsDNA ends [84]. Some reports indicate reduced viability of *uvrD recB* mutant strains [91] whereas others report inviability [92,93]. We assayed the viability of Δ*uvrD recB^−^* *rpoB^+^* cells by analysing their ability to lose pRC7*recB*. Δ*uvrD recB^−^* *rpoB^+^* cells could generate white colonies on rich medium in contrast to Δ*rep recB^−^* *rpoB^+^* cells (Figure 5, compare B with C). However, the frequency of Δ*uvrD recB^−^* *rpoB^+^* white colony formation was lower and white colony sizes much smaller than with *uvrD^+^ recB^−^* *rpoB^+^* cells (Figure 5, compare B with A). Thus, in this strain, background cells can survive without both UvrD and RecBCD but growth is impaired.

**Figure 6:**
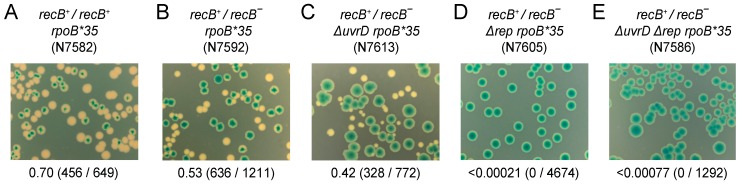
RecB is essential in rep uvrD rpoB*35 cells on rich medium. (**A**–**E**) Loss of pAM375 (pRC7*recB*) from the strains indicated was monitored on LB Xgal IPTG.

**Figure 7:**
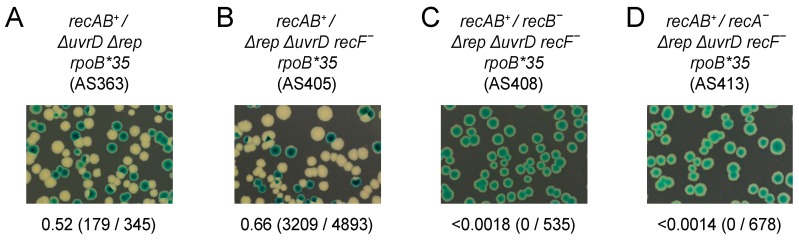
The requirement for RecBCD and RecA is not alleviated by mutation of *recF*. (**A**–**D**) Loss of pAM406 (pRC7*recA*, *recB*) from the strains indicated was monitored on LB Xgal IPTG.

*rpo* mutations that suppress Δ*rep* Δ*uvrD* double mutant lethality are unable to suppress Δ*rep recB* double mutant lethality [78]. Similarly, reduction of transcription-driven replicative barriers using rpoB*35 did not improve the viability of either Δ*rep recB* or Δ*uvrD recB* double mutant strains (compare Figure 6C with Figure 5B; Figure 6D with Figure 5C). Δ*rep* ΔuvrD rpoB*35 *recB^−^* was also inviable (Figure 6E), as expected given the growth defects of single *rep* and *uvrD* mutants [71] (Figure 6C,D). It was possible that UvrD not being available to abort RecFOR-directed loading of RecA onto blocked replication forks [81,84] contributed to Δ*rep* ΔuvrD rpoB*35 *recB* inviability. However, Δ*rep* ΔuvrD rpoB*35 recF^−^ *recB^−^* remained inviable, indicating that countering RecFOR activity was not a major contributor to this inviability (Figure 7A–C). Δ*rep* ΔuvrD rpoB*35 recF^−^ recA^−^ also remained inviable (Figure 7D), as expected given that RecFOR-dependent toxicity requires RecA [84].

These data indicate that RecA (Figure 4B and Figure 7D) and RecBCD (Figure 6E and Figure 7C) are both essential in Δ*rep* Δ*uvrD* mutant cells under rapid growth conditions, even when replication-transcription conflicts are reduced by a mutation in RNA polymerase. Thus when accessory helicases are absent the degradation of double-stranded DNA ends by RecBCD is insufficient by itself to deal with blocked replication forks. Under such circumstances strand exchange is also needed, allowing D-loop formation from double-stranded DNA ends and subsequent replisome reloading [1,94].

## 4. Discussion

We show here that transcription is a major cause of the chromosomal degradation seen in *recA* cells. The chromosome content defects of cells lacking either Rep [6] or RecA (Figure 1A, compare (iii) with (vii)) share the same primary cause therefore indicating that both Rep and RecA reduce the impact of gene expression on genome duplication. The synergistic increase in chromosome content defects in *rep* *recA* cells indicate that these enzymes provide alternative means of mitigating the impact of transcription on DNA replication (Figure 1A (iv)). Furthermore, the significant suppression of DNA degradation in *recA* cells by an RNA polymerase mutation supports the view that protein-DNA complexes are the primary causes of replication defects in cells not exposed to elevated DNA damage [6]. However, the time taken to duplicate a chromosome is not extended in the absence of either RecA or RecB, in contrast to cells lacking Rep (Figure 3). Thus the maintenance of rapid chromosome duplication has a greater dependency on Rep as opposed to RecA or RecBCD. RecA and RecBCD do, though, have the ability to sustain chromosome duplication in Δ*rep* Δ*uvrD* double mutant cells when transcriptional barriers to replication are reduced (Figure 4 and Figure 7). Both RecA and RecBCD are needed for this underpinning, demonstrating that maintenance of chromosome duplication by recombination enzymes is most efficient when RecBCD catalyses loading of RecA at dsDNA ends rather than large-scale RecBCD-dependent degradation of such ends (Figure 7).

**Figure 8:**
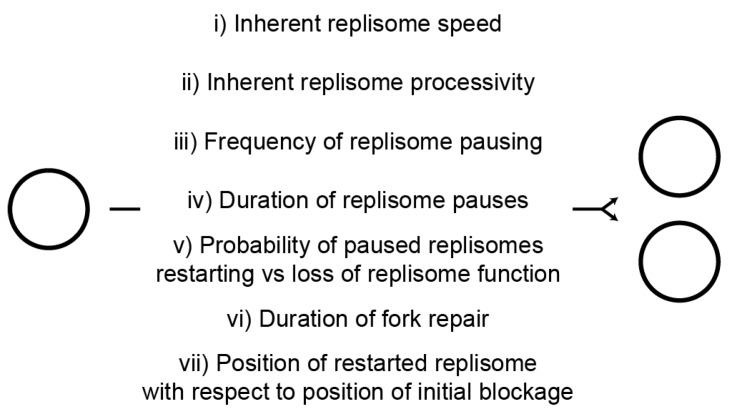
Summary of factors with potential influence on the probability of replisomes completing chromosome duplication and the time needed to do so.

These data are apparently contradictory. RecA has little impact on chromosome duplication times and, although the time resolution of the measurements in Figure 3 are relatively low, they still imply infrequent engagement of RecA in genome duplication during a single cell cycle. In contrast, the absence of RecA results in frequent chromosome degradation [55] and a reduction in viability [85]. In considering this apparent contradiction, there are many factors that potentially affect the time needed to copy a chromosome (Figure 8). The inherent speed and processivity of the replisome is an important determinant of chromosome duplication time but the pausing behaviour of replisomes, and what happens to these paused forks, will also impact on duplication times (Figure 8 (i–v)). Accessory helicases reduce the frequency and/or duration of replisome pauses at nucleoprotein complexes and increase the probability of paused replisomes restarting replication as opposed to losing function [6,22,27]. The extended chromosome duplication time in Δ*rep* mutant cells [29,31] indicates that one or more of these pausing parameters are critical in determining the speed of chromosome duplication. The more than twofold increase in chromosome duplication times in Δ*rep* mutant cells probably also underrepresents the significance of replisome pausing behaviour on these timings, given the ability of UvrD to compensate partially for the absence of Rep [22,30].

Regardless of the cause of replisome pausing, there is no evidence that recombination enzymes impact directly on paused replisomes but they can act after a replisome has lost function to promote replication restart [7]. Loss of enzymes such as RecA or RecBCD might therefore impact on the duration of any fork repair process since loss of an enzyme normally involved in fork repair might lead to extension of repair times due to less efficient alternative pathways that are not normally operative in wild type cells (Figure 8 (vi)). A related consideration is the position of replication re-initiation with respect to the position of the blocked initial replisome (Figure 8 (vii)). The extensive RecBCD-dependent degradation of DNA in *recA* mutant cells [55] argues for less efficient replication repair for both of the above reasons. Firstly, the time taken to degrade extensive sections of the chromosome is measured in minutes even with the high speed and processivity of RecBCD-catalysed dsDNA end degradation [56]. Secondly, this extensive degradation in effect means that replisome reloading must occur far upstream of the initially blocked fork, possibly at *oriC* [95]. However, the absence of significant extension of chromosome duplication time in *recA* or *recB* mutant cells (Figure 3) indicates that fork repair in the absence of either activity does not impact significantly on the mean duplication time during a single cell cycle. The reduced viability [85] and chromosomal degradation seen in *recA* mutant cells [55] (and Figure 1A (iii)) might therefore reflect the loss of replisome function when considering multiple cell cycles rather than just one. These occasional repair events may be too infrequent to have a measurable impact on mean chromosome duplication time during one cell cycle (Figure 3) but each event might take significant time and result in accumulation of cells with different numbers of chromosome equivalents over the course of multiple cell cycles. Given enough time, though, these non-wild type repair events can resolve the majority of replicative problems, evinced by the similar chromosome profiles of wild type and *recA* mutant cells in stationary phase (Figure 2). Regarding reduced viability of *recA* mutant cells, such viability measurements involve comparing the number of colony-forming units with the total number of cells as determined by microscopy [85]. This measure of viability therefore indicates the relative frequency with which a population of cells generates non-viable cells over the course of an extended period of time and cannot be compared to a measure of chromosome duplication time during a single cell cycle as presented in Figure 3. Infrequent engagement of RecA and RecBCD during chromosome duplication might have an undetectable impact on the mean time taken to replicate a chromosome during a single cell cycle. However, absence of RecA- and RecBCD-dependent processing of replication intermediates could result in aberrant events in their absence which, over multiple cell cycles, gives rise to cells that can no longer divide.

The inefficiency of non-wild type fork repair mechanisms might also relate to our finding that Δ*rep* ΔuvrD rpoB*35 recF^−^ cells require both RecA and RecBCD for survival (Figure 4 and Figure 7). *rep recA* double mutant cells are viable, but *rep recB* double mutants are not, indicating that under some circumstances RecBCD-catalysed degradation of dsDNA ends in the absence of RecA can underpin genome duplication [58,71]. However, our data indicate that when both Rep and UvrD are absent then RecBCD-dependent DNA degradation is not sufficient to sustain viability unless it is coupled to loading of RecA. UvrD can act as an accessory helicase and compensate partially for loss of Rep in Δ*rep* *uvrD^+^* cells [22,30]. This partial compensation may explain why DNA degradation without RecA loading can maintain viability in Δ*rep* *uvrD^+^* cells but not in Δ*rep* ΔuvrD rpoB*35 recF^−^ cells: dsDNA end degradation alone provides an inefficient means of reinitiating DNA replication if replisomes pause and lose function at an elevated rate, as in cells lacking both Rep and UvrD.

It should also be borne in mind that, whilst transcription is a major source of replicative problems in unstressed cells [6,22,30,78] (Figure 1), recombination enzymes have the ability to deal with replicative barriers other than protein-DNA complexes, unlike accessory replicative helicases [1,7]. Thus under conditions of elevated replicative stress such as exogenous DNA damaging agents then recombination enzymes may dominate replication repair. However, in otherwise unstressed cells our data are consistent with the accessory helicase-dependent minimisation of replisome pausing having a more significant impact on sustaining replisome movement than recombination-directed replisome reloading mechanisms.

## 5. Conclusions

Rep and RecA provide alternative means of mitigating the impact of transcription on genome duplication but the maintenance of rapid genome duplication is more dependent on Rep than RecA. In the absence of accessory replicative helicase activity (Δ*rep* Δ*uvrD* cells carrying rpoB*35) both RecA and RecBCD are needed to maintain viability. Thus when fork pausing at protein-DNA complexes is very frequent RecBCD-dependent degradation of DNA ends is insufficient for survival and RecBCD nuclease activity must be coupled to loading of RecA. We conclude that Rep is important to ensure rapid movement of the majority of replication forks from origin to terminus and that RecABCD are needed for a subset of replicative problems that are otherwise difficult to deal with.

## Supplementary Materials

The following are available online at www.mdpi.com/2073-4425/7/8/42/s1, Table S1: *Escherichia coli* K12 strains.
