# Supplementary material for: The Balance between Recombination Enzymes and Accessory Replicative Helicases in Facilitating Genome Duplication
Source: Genes (Basel). 2016 Jul 29;7(8):42. doi: 10.3390/genes7080042 (PMC4999830; doi:10.3390/genes7080042)
Supplement: Supplementary file 1 [file genes-07-00042-s001.docx]

Supplementary Materials: The Balance between Recombination Enzymes and Accessory Replicative Helicases in Facilitating Genome Duplication

Aisha H. Syeda, John Atkinson, Robert G. Lloyd and Peter McGlynn

**Table S1.** *Escherichia coli* K12 strains.

| **(A) MG1655 Derivatives** | | |
| --- | --- | --- |
| MG1655 | F^−^ rph-1 | [1] |
| AM1573 | ΔlacIZYA recB270::kan | P1.RJ1003 × TB28 to Km^r^ |
| AM1590 | pAM375 (lac^+^ recB^+^)/ΔlacIZYA Δrep::cat recB268::kan | [2] |
| AM1657 | ΔuvrD::dhfr | [3] |
| AM2158 | ΔlacIZYA rpoB[G1260D] | [4] |
| AS301 | ΔlacIZYA rpoB*35 ΔuvrD::dhfr Δrep::cat | Plasmid-free segregant of N7150 |
| AS351 | pAM383 (lac^+^ recA^+^)/ΔrecA::spec | pAM383 × N7358 to Ap^r^ |
| AS363 | pAM406 (lac^+^ recA^+^ recB^+^)/ΔlacIZYA rpoB*35 ΔuvrD::dhfr Δrep::cat | pAM406 × AS301 to Ap^r^ |
| AS370 | pAM406 (lac^+^ recA^+^ recB^+^)/ΔlacIZYA rpoB*35 ΔuvrD::dhfr Δrep::cat *recB268::*Tn*10* | P1.BP45 × AS363 to Tc^r^ |
| AS371 | pAM406 (lac^+^ recA^+^ recB^+^)/ΔlacIZYA rpoB*35 ΔuvrD::dhfr Δrep::cat ΔrecA::spec | P1.AS351 × AS363 to Spec^r^ |
| AS405 | pAM406 (lac^+^ recA^+^ recB^+^)/ΔlacIZYA rpoB*35 ΔuvrD::dhfr Δrep::cat ΔrecF735::<kan> | P1.JW3677 × AS363 to Km^r^ |
| AS408 | pAM406 (lac^+^ recA^+^ recB^+^)/ΔlacIZYA rpoB*35 ΔuvrD::dhfr Δrep::cat ΔrecF735::<kan> *recB268::*Tn*10* | P1.JW3677 × AS370 to Km^r^ |
| AS413 | pAM406 (lac^+^ recA^+^ recB^+^)/ΔlacIZYA rpoB*35 ΔuvrD::dhfr Δrep::cat ΔrecF735::<kan> ΔrecA::spec | P1.JW3677 × AS371 to Km^r^ |
| BP45 | *∆ara714 argEC::[apra^r^ lacO_34_] recB268::*Tn*10* | [5] |
| HB159 | ΔlacIZYA dnaA46 tna300::Tn10 | [6] |
| HB161 | ΔlacIZYA dnaA46 tna300::Tn10 Δrep::cat | [6] |
| HB278 | ΔlacIZYA rpoB[G1260D] Δrep::cat | [7] |
| HB310 | ΔlacIZYA rpoB[G1260D] Δrep::cat recA269::Tn10 | P1.N4279 × HB278 toTc^r^ |
| HB312 | ΔlacIZYA rpoB[G1260D] recA269::Tn10 | P1.N4279 × AM2158 toTc^r^ |
| JA042 | ΔlacIZYA dnaA46 tna300::Tn10 ΔrecA::kan | P1.N6618 × HB159 to Km^r^ |
| JA044 | ΔlacIZYA dnaA46 tna300::Tn10 recB270::kan | P1.N4600 × HB159 to Km^r^ |
| N4279 | recA269::Tn10 | [8] |
| N4600 | recB270::kan | P1.RJ1003 × MG1655 |
| N5925 | ΔlacIZYA rpoB*35 | [4] |
| N5988 | pAM375 (lac^+^ recB^+^)/ΔlacIZYA recB270::kan | pAM375 × AM1573 to Ap^r^ |
| N6065 | ΔlacIZYA recA269::Tn10 | [2] |
| N6524 | pAM403 (lac^+^ rep^+^)/ΔlacIZYA | [4] |
| N6540 | pAM403 (lac^+^ rep^+^)/ΔlacIZYA Δrep::cat | P1.JJC735 × N6524 to Cm^r^ |
| N6577 | ΔlacIZYA Δrep::cat | [4] |
| N6618 | ΔrecA::kan | This work |
| N7150 | pAM407 (lac^+^ uvrD^+^)/ΔlacIZYA rpoB*35 ΔuvrD::dhfr Δrep::cat | [4] |
| N7153 | ΔlacIZYA rpoB*35 ΔuvrD::dhfr Δrep::cat | Plasmid-free segregant of N7150 |
| N7358 | ΔrecA::spec | [9] |
| N7578 | pAM407 (lac^+^ uvrD^+^)/ΔlacIZYA rpoB*35 ΔuvrD::dhfr Δrep::cat recA269::Tn10 | P1.N3072 × N7150 to Tc^r^ |
| N7581 | pAM375 (lac^+^ recB^+^)/ΔlacIZYA rpoB*35 ΔuvrD::dhfr Δrep::cat | pAM375 × N7153 to Ap^r^ |
| N7582 | pAM375 (lac^+^ recB^+^)/ΔlacIZYA rpoB*35 | pAM375 × N5925 to Ap^r^ |
| N7586 | pAM375 (lac^+^ recB^+^)/ΔlacIZYA rpoB*35 recB268::Tn10 ΔuvrD::dhfr Δrep::cat | P1.TRM308 × N7581 to Tc^r^ |
| N7592 | pAM375 (lac^+^ recB^+^)/ΔlacIZYA rpoB*35 recB268::Tn10 | P1.TRM308 × N7582 to Tc^r^ |
| N7602 | pAM403 (lac^+^ rep^+^)/ΔlacIZYA Δrep::cat recA269::Tn10 | P1.N3072 × N6540 to Tc^r^ |

**Table S1.** *Cont.*

| **(A) MG1655 Derivatives** | | |
| --- | --- | --- |
| N7603 | ΔlacIZYA Δrep::cat recA269::Tn10 | Plasmid-free segregant of N7602 |
| N7605 | pAM375 (lac^+^ recB^+^)/ΔlacIZYA rpoB*35 recB268::Tn10 Δrep::cat | P1.JJC735 × N7592 to Cm^r^ |
| N7613 | pAM375 (lac^+^ recB^+^)/ΔlacIZYA rpoB*35 recB268::Tn10 ΔuvrD::dhfr | P1.AM1657 × N7592 to Tm^r^ |
| RJ1003 | relA1 ΔspoT207:cat rpoB*35 ΔruvAC65 eda-51::Tn10 recB270::kan | [10] |
| SW1093 | pAM375 (lac^+^ recB^+^)/ΔlacIZYA recB270::kan ΔuvrD::dhfr | P1.AM1657 × N5988 to Tm^r^ |
| TB28 | ΔlacIZYA | [11] |
| TRM308 | recB268::Tn10 sbcA | [2] |
| **(B) Other Derivatives** | | |
| JW3677 | BW25113 rrnB3 *∆*lacZ4787 hsdR514 *∆*(araBAD)567 *∆*(rhaBAD)568 rph-1 ΔrecF735::<kan> | [12] |
| JJC735 | AB1157 *hsdR* Δrep::cat | [13] |
| N3072 | W3110 rph-1 IN(rrnD-rrnE)1 recA269::Tn10 | [14] |

References

1. Bachmann, B.J. Derivations and genotypes of some mutant derivatives of *Escherichia coli* K-12. In *Escherichia coli and Salmonella Cellular and Molecular Biology*, 2nd ed.; Neidhardt, F.C., Curtiss, R., III; Ingraham, J.L., Lin, E.C.C., Low, K.B., Magasanik, B., Reznikoff, W.S., Riley, M., Schaechter, M., Umbarger, H.E., Eds.; ASM Press: Washington, DC, USA, 1996; pp. 2460–2488.
2. Mahdi, A.A.; Buckman, C.; Harris, L.; Lloyd, R.G. Rep and PriA helicase activities prevent RecA from provoking unnecessary recombination during replication fork repair. *Genes Dev.* **2006**, *20*, 2135–2147.
3. Zhang, J.; Mahdi, A.A.; Briggs, G.S.; Lloyd, R.G. Promoting and avoiding recombination: Contrasting activities of the *Escherichia coli* RuvABC Holliday junction resolvase and RecG DNA translocase. *Genetics* **2010**, *185*, 23–37.
4. Guy, C.P.; Atkinson, J.; Gupta, M.K.; Mahdi, A.A.; Gwynn, E.J.; Rudolph, C.J.; Moon, P.B.;
   van Knippenberg, I.C.; Cadman, C.J.; Dillingham, M.S.; et al. Rep provides a second motor at the replisome to promote duplication of protein-bound DNA. *Mol. Cell* **2009**, *36*, 654–666.
5. Payne, B.T.; van Knippenberg, I.C.; Bell, H.; Filipe, S.R.; Sherratt, D.J.; McGlynn, P. Replication fork blockage by transcription factor-DNA complexes in *Escherichia coli*. *Nucleic Acids Res.* **2006**, *34*, 5194–5202.
6. Atkinson, J.; Gupta, M.K.; Rudolph, C.J.; Bell, H.; Lloyd, R.G.; McGlynn, P. Localization of an accessory helicase at the replisome is critical in sustaining efficient genome duplication. *Nucleic Acids Res.* **2011**, *39*, 949–957.
7. Gupta, M.K.; Guy, C.P.; Yeeles, J.T.; Atkinson, J.; Bell, H.; Lloyd, R.G.; Marians, K.J.; McGlynn, P.
   Protein-DNA complexes are the primary sources of replication fork pausing in *Escherichia coli*. *Proc. Natl. Acad. Sci. USA* **2013**, *110*, 7252–7257.
8. McGlynn, P.; Lloyd, R.G. Modulation of RNA polymerase by (p)ppGpp reveals a RecG-dependent mechanism for replication fork progression. *Cell* **2000**, *101*, 35–45.
9. Briggs, G.S.; Yu, J.; Mahdi, A.A.; Lloyd, R.G. The RdgC protein employs a novel mechanism involving a finger domain to bind to circular DNA. *Nucleic Acids Res.* **2010**, *38*, 6433–6446.
10. Trautinger, B.W.; Jaktaji, R.P.; RUSAkova, E.; Lloyd, R.G. RNA polymerase modulators and DNA repair activities resolve conflicts between DNA replication and transcription. *Mol. Cell* **2005**, *19*, 247–258.
11. Bernhardt, T.G.; de Boer, P.A. Screening for synthetic lethal mutants in *Escherichia coli* and identification of EnvC (YibP) as a periplasmic septal ring factor with murein hydrolase activity. *Mol. Microbiol.* **2004**, *52*, 1255–1269.
12. Baba, T.; Ara, T.; Hasegawa, M.; Takai, Y.; Okumura, Y.; Baba, M.; Datsenko, K.A.; Tomita, M.; Wanner, B.L.; Mori, H. Construction of *Escherichia coli* K-12 in-frame, single-gene knockout mutants: The Keio collection. *Mol. Syst. Biol.* **2006**, doi:10.1038/msb4100050.
13. Bidnenko, V.; Seigneur, M.; Penel-Colin, M.; Bouton, M.F.; Dusko Ehrlich, S.; Michel, B. *sbcB sbcC* null mutations allow RecF-mediated repair of arrested replication forks in *rep recBC* mutants. *Mol. Microbiol.* **1999**, *33*, 846–857.
14. Picksley, S.M.; Lloyd, R.G.; Buckman, C. Genetic analysis and regulation of inducible recombination in *Escherichia coli* K-12. *Cold Spring Harb. Symp. Quant. Biol.* **1984**, *49*, 469–474.

© 2016 by the authors. Submitted for possible open access publication under the
terms and conditions of the Creative Commons Attribution (CC-BY) license (http://creativecommons.org/licenses/by/4.0/).
